# Recovery trajectories after a serious injury or illness: a longitudinal evaluation of health-related quality of life in an Australian cohort

**DOI:** 10.1007/s11136-025-03919-w

**Published:** 2025-02-17

**Authors:** Clifford Afoakwah, Paul Kuwornu, Isaac Koomson, David Brain, Qing Xia, Steven McPhail, Kirsten Vallmuur

**Affiliations:** 1 Australian Centre for Health Services Innovation, School of Public Health and Social Work, Queensland University of Technology, Kelvin Grove, QLD Australia; 2 Jamieson Trauma Institute, Metro North Health, Herston, QLD Australia; 3 Centre for the Business and Economics of Health, The University of Queensland, St Lucia, QLD Australia

**Keywords:** Serious injury or illness, Recovery, Quality-adjusted life years, physical health, Mental health

## Abstract

**Purpose:**

Serious injuries or illnesses impose a significant burden on the affected individuals. This study examined the long-term recovery of health-related quality of life (HRQoL) after a serious injury or illness and quantified the economic costs attributable to the quality-adjusted life-years (QALYs) lost to serious injury or illness.

**Methods:**

Data were sourced from the Household, Income and Labour Dynamics in Australia (HILDA) database. The propensity score matching-difference-in-differences (PSM-DiD) method was used to explore the recovery of HRQoL over a six-year follow-up period. HRQoL was measured by the short-form six-dimension utility index, physical health, and mental health.

**Results:**

We found that those who suffered any serious injuries or illnesses had a decline of 0.020 in QALYs during the year of event, reducing further until a trough of 0.032 by the third year and thereafter began to recover. Further analyses revealed that although serious injuries or illnesses had a significantly greater loss in physical health, the loss in mental health persisted over a longer period. Finally, the economic cost associated with the lost in QALYs due to serious injuries or illnesses was estimated at $685 per person during the year of event and increased to a peak of $1,250 per person by the end of the third-year post-exposure.

**Conclusion:**

Our findings highlight that follow-up care designed to mitigate the impacts of a serious injury or illness on people’s HRQoL should consider cost-effective strategies that are long-lasting and support those affected throughout at least, the first three years of their injuries or illnesses.

**Supplementary Information:**

The online version contains supplementary material available at 10.1007/s11136-025-03919-w.

## Introduction

Serious injuries or illnesses can severely compromise one’s quality of life, increase the strains on one’s caregivers and put one at high risk of death [1]. Such health conditions, whether chronic or acute, often require immediate treatment as an in-patient in a hospital and involve incapacity for more than three days with continuing treatment by a healthcare provider. Personal serious injury or illness, therefore, imposes a significant burden on the affected persons, health system and society at large. Serious injuries, for example, remain a major health issue in Australia accounting for 8.1% of the burden of disease, 7.6% of health expenditure, and the leading cause of death among individuals under the age of 45 years [2]. Similarly, serious chronic conditions such as mental and behavioural conditions, back problems, arthritis, diabetes, heart, stroke and vascular disease, osteoporosis, chronic obstructive pulmonary disease, cancer, and kidney disease affect one in two people in Australia [3].

Duration-wise, serious injury or illness can negatively impact one’s health-related quality of life (HRQoL) both in the short and long term. Although mortality remains the most common outcome studied, recent studies have incorporated the HRQoL of trauma survivors as most patients survive their health shock [4]. Serious injury or illness may result in varying degrees of disability, which may have several social and/or economic consequences, such as the affected person becoming unable to return to work, regain their previous levels of physical activity, or reintegrate into their social circles [5]. Consequently, patient-reported outcome measures such as the HRQoL have become predominant because of their ability to integrate the consequences of a serious injury or illness into the various dimensions of the person’s life.

Hence, examining a broader population who have experienced any serious injury or illness and comparing their outcomes before and after the illness with those who did not have any serious injury or illness provides an opportunity to investigate the recovery of overall HRQoL as well as the relative contribution of both physical and mental factors to HRQoL [6]. While most studies report that individuals who suffer serious injury or illness experience a compromised HRQoL [7, 8], the follow-up periods are very short (6 to 24 months) or without comparators to estimate the counterfactual HRQoL of those who had a serious injury or illness. More so, previous studies mostly investigated the impacts of serious injury or illness on selected domains of HRQoL (such as physical or mental health), but not on overall health state utilities. Health state utilities are used to provide quantitative measures of a person’s preference for specific states of health on a scale where 0 represents death and 1 represents perfect health, with values less than 0 representing states worse than death [9]. Given their importance in health economic evaluations, health technology assessments, and reimbursement decision-making, unpacking the impacts of a serious injury or illness on health state utility scores and QALYs is of great value.

The overarching aim of this study is to examine the effect of a serious injury or illness on HRQoL over time and quantify the economic costs attributable to the quality-adjusted life-years (QALYs) lost due to an injury or illness. By doing so, our study makes valuable contributions to the literature. Firstly, our quasi-experimental study design, backed by a rich dataset, allows us to evaluate the year-on-year recovery pathways after a serious injury or illness. Specifically, our propensity score matching-difference in differences (PSM-DiD) analytical strategy enables comparison of HRQoL before and after a serious injury or illness for both exposed and unexposed groups. Previous studies have included only post-illness data [8, 10], without comparators [7], or short-term follow-up period [6], making it difficult to evaluate the long-term trajectory of a person’s HRQoL after a serious injury or illness. Secondly, our nuanced analyses of both physical and mental health impacts after a serious health event provide useful information for designing tailored policies to improve the different domains of a person’s HRQoL. Finally, unlike previous studies, our study proceeds to estimate, for the first time, the intrinsic value (economic cost) of QALYs lost due to a serious injury or illness. The indirect economic impacts of injuries or illnesses include not only lost productivity, reduced earnings, and long-term disability, but also diminished quality of life. While the current literature predominantly focuses on quantifying lost productivity and earnings and long-term disability [11–13], the quantification of the intrinsic value of QALYs lost due to a serious injury or illness remains underexplored. Such information is critical and will serve as a benchmark for the economic evaluation of health interventions- whether models of care or treatment strategies- that seek to improve the recovery and HRQoL for those who have suffered any serious injuries or illnesses.

## Methods

### Data

This study used secondary data from the Household, Income and Labour Dynamics in Australia (HILDA) survey. The HILDA survey collects information on the economic and personal well-being, labour market dynamics and family life across households over time in Australia. The survey began in 2001 and follows more than 17,000 Australians each year. This study used version 21, and the cohort identification year was 2014. We chose 2014 as the cohort identification year because we wanted a contemporary cohort and needed to follow individuals for at least five years while avoiding any contamination effect from the COVID-19 pandemic that began in December 2019.

### Health-related quality of life measures

We measured HRQoL using the Short Form Six Dimension (SF-6D), physical functioning and mental health derived from 36-Item Short Form Survey (SF-36) quality of life instruments [14, 15]. The SF-36 consist of 36 questions that are grouped into eight domains of health and well-being: general health, mental health, physical function, bodily pain, role physical, vitality, social function, and role emotion. It is not a disease-specific instrument and, therefore, enables comparison across different diseases. During the survey, respondents were asked to complete the SF-36 questionnaire and the responses were standardized with a scoring algorithm to obtain values ranging from zero (worst health state) to 100 (best health state) [15], with a unit increase in the score reflecting an improvement in the outcome it captures. This study focused on only two domains of the SF-36 instruments: physical functioning (hereafter referred to as physical health) and mental health, as these domains can directly be impacted by a serious injury or illness.

To have a single measure that encapsulates the different health domains of a person, we also analysed the SF-6D, which is a generic health utility measure that was derived from the SF-36 and used to value health services in terms of quality-of-life improvement. It consists of eleven questions from the SF-36 instrument and is divided into six health domains: bodily pain, mental health, physical functioning, social functioning, role-limitation (combined role-physical and role emotional), and vitality [5]. The SF-6D allows the capture of HRQoL as the manifestation of different health outcomes in a single index [15], although it can be sensitive to how preference weights are mapped from the SF-36 instrument [16]. The SF-6D responses were then transformed to utility values using the Australian utility weights and ranged from zero (worst health state) to one (best health state). The coefficients from the SF-6D regressions were converted to QALYs (by multiplying the coefficients by time spent in that health state, which is one year, except for the 6-year total effect model) and interpreted as such.

### Exposure variable

**Fig. 1:**
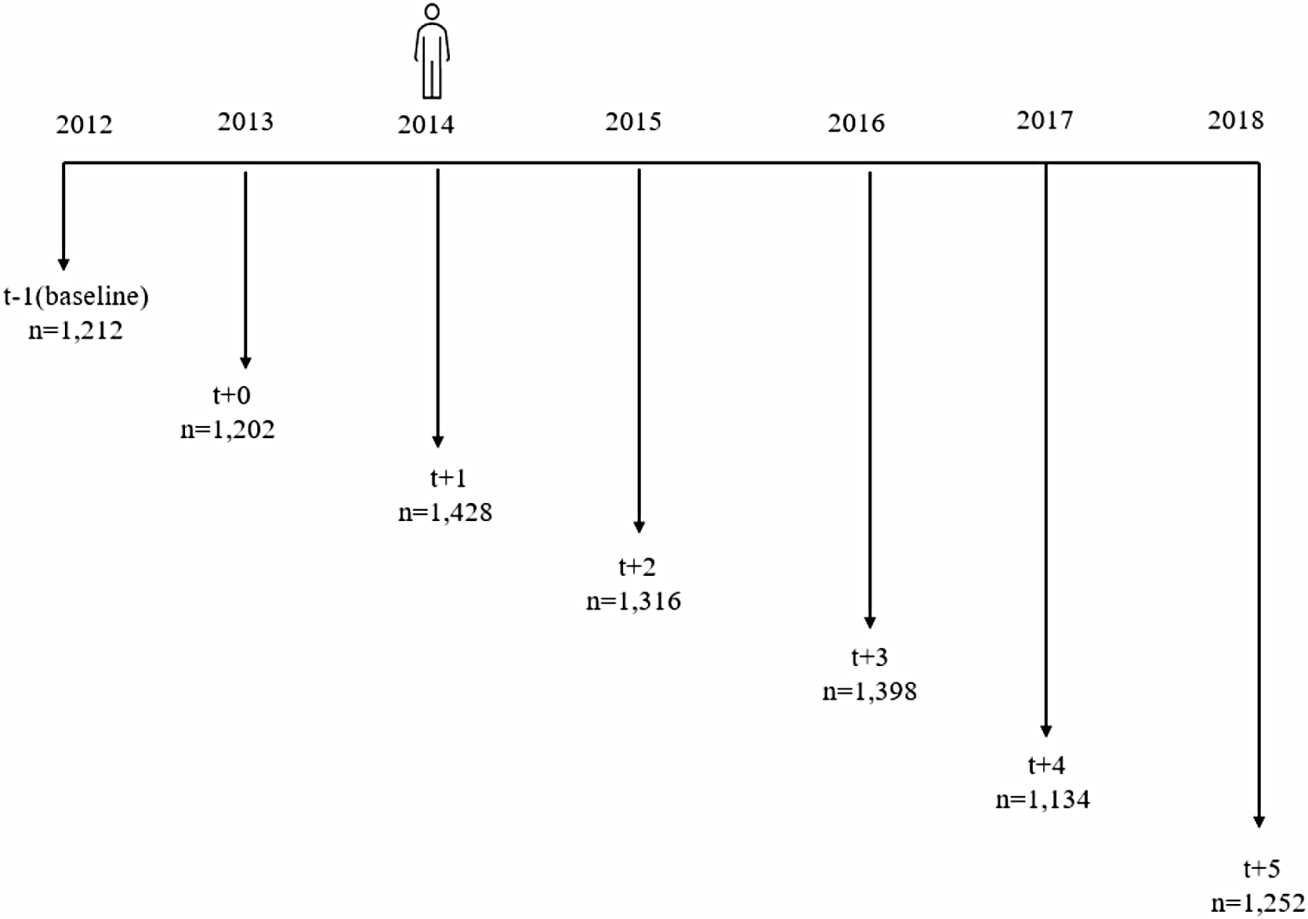
Cohort identification and follow-up periods

The exposure variable in this study is whether an individual had any serious injury or illness. During the survey, respondents aged 15 years and above were asked whether they have had any personal serious injury or illness in the past twelve months preceding the survey. Since this variable is a recall of an event that happened at most 12 months prior and considering that the survey was conducted in July 2014, we assumed 2013 to be the year the event occurred, 2012 as the year before the injury or illness and 2014 as one-year post-illness. The subsequent follow-up periods are defined in Fig. 1.

### Analytical strategy

The propensity score matching-difference in differences (PSM-DiD), which is a two-step analytical strategy, was utilised to examine the recovery trajectories of HRQoL. Since this study employs a quasi-experimental design, the propensity score matching (PSM) approach was first employed to generate an unexposed group, as it can address any selection bias (due to observable attributes) that might compromise our estimates [17]. That is, it is possible that the occurrence of a serious injury or illness is not random and might be influenced by some observable sociodemographic characteristics and one’s prior health status. We used the PSM to match individuals who suffered any serious injury or illness (hereafter referred to as the exposed group) to those who did not experience any serious injury or illness (hereafter referred to as the unexposed group) based on their propensity scores. The one-to-one nearest neighbour matching without replacement was used to generate matched pairs. Matching was performed using pre-exposure characteristics, including age (15–24, 25–44, 45–64, and 65 and above), sex, education levels (high school and below, diploma, and bachelor’s degree and above), and HRQoL (including the SF-6D, physical health and mental health). These variables have been shown to be key determinants of a serious injury or illness [18, 19]. The standardised mean difference (SMD) statistic was used to determine the balance of covariate distribution between the two groups, with values of 0.1 or below deemed to be a good balance.

After matching was performed, the DiD approach was used to estimate the impact of a serious injury or illness on HRQoL at six time periods: year of exposure (t + 0), one year post-exposure (t + 1), two years post-exposure (t + 2), three years post-exposure (t + 3), four years post-exposure (t + 4), and five years post-exposure (t + 5). The DiD addresses any selection bias resulting from unobservable confounders. The DiD regression is of the following specification:

1 $$\begin{aligned} HRQo{L_{it}} & ={\beta _1}+{\beta _2}Expose{d_i}+{\beta _3}Pos{t_t} \\ & +{\beta _4}\left( {Expose{d_i} \times ~Pos{t_t}} \right)+X_{{it}}^{\prime }\partial +{\varepsilon _{it}} \\ \end{aligned} $$

Where $\:HRQoL$ is HRQoL of individual $\:i$ at time $\:t$ and proxied with three measures: SF-6D utility scores, physical health, and mental health from the SF-36 instruments. $\:Exposed$ is the exposure variable which is measured as 1 if individual $\:i$ had a serious injury or illness and zero if otherwise. $\:Post$ is a time dummy which is equal to 1 and 0 for post and pre-exposure periods, respectively. $\:{Exposed}_{i}*\:{Post}_{t}$ is the DiD term, which is the interaction term for the exposure status and time variables, hence $\:{\beta\:}_{4}$ is our coefficient of interest. $\:{X}^{{\prime\:}}$ is a vector of time-variant and time in-variant control variables, including sex, age groups, marital status, educational status, location of usual residence (remote, regional, or major city) and body mass index that has shown to impact on a person’s HRQoL [20–22]. The average treatment effect on the treated (ATT) was interpreted as the effect of a serious injury or illness on HRQoL. Individuals with missing information on HRQoL during any of the study years were excluded from the analysis, hence we utilised an intention-to-treat analytical approach.

Finally, the economic cost of a serious injury or illness was computed using a back-of-the-envelope approach, where QALY loss was multiplied by willingness-to-pay (WTP) per QALY estimate of $42,000 [23].The $50,000 WTP threshold for public funding of new health technologies in the Australian health system [24] was also applied for sensitivity test. All costs and 95% confidence intervals (CIs) were reported as per person and in Australian dollars. Since the WTP per QALY estimate reflects 2018 values, we adjusted for inflation for our study years using the health group index (as suggested by Afoakwah et al. [25]) sourced from the Australian Bureau of Statistics [26]. QALYs were not discounted as they were observed in their respective years under consideration [27].

## Results

### Cohort identification and characteristics

Before matching, the exposed group were 1,428 individuals (Fig. 1). 84% (1,202) of this cohort could be tracked in 2013, while 84.9% (1,212) were identified during the 2012 survey. Prospectively, 92.2% of the cohort were followed up for one year, 97.9% for two years, 79.4% for three years and 87.7% for four years (Appendix A). Since matching was performed using the baseline covariates, only individuals captured during the 2012 survey constituted the exposed and unexposed groups.

The characteristics of the cohort before and after matching are presented in Table 1. There were significant differences in sociodemographic characteristics (gender, age, educational levels) and HRQoL between the exposed and unexposed groups before matching. However, these differences disappeared after matching was performed as gender, age educational level and HRQOL were found to be balanced with no significant difference between both exposed and unexposed groups, as evidenced by the between-group SMD.

**Table 1:** Baseline characteristics between exposed and control group before and after PSM

	Before PSM			After PSM			
Variables	Exposed N(SF6D) = 8,942 N(PH = 9,272) N(MH) = 9,319	Control N(SF6D) = 89,362 N(PH) = 91,725 N(MH) = 92,226	SMD	Exposed N(SF6D) = 7,638 N(PH) = 8144 N(MH) = 8,186	Control N(SF6D) = 7,638 N(PH) = 8144 N(MH) = 8,186	SMD	
**Female** (%)	51.37	53.97	0.052	51.41	50.69	-0.014	
**Age** (%)							
15 to 24	7.26	11.91	0.159	7.27	7.35	0.003	
25–44	22.62	34.03	0.255	22.64	22.81	0.004	
45–64	37.11	35.43	-0.035	37.13	37.03	-0.002	
65 and above	33.01	18.63	-0.333	32.97	32.82	-0.003	
**Education** (%)							
Year 12 and below	39.96	38.83	-0.023	39.96	39.62	-0.007	
Diploma	36.57	32.67	-0.082	36.57	36.87	0.006	
Bachelor’s and above	23.47	28.5	0.115	23.47	23.47	0.001	
**Pre-exposure** HRQoL							
SF-6D	0.699	0.772	0.582	0.699	0.699	0.001	
PH	72.67	85.82	0.543	72.67	74.15	0.054	
MH	70.58	75.26	0.259	70.57	70.13	-0.023	

MH: mental health; PH: physical health. PSM: propensity score matching; SMD: standardized mean differences

### Serious injury or illness and health-related quality of life

Appendix B shows that mean HRQoL of the exposed group decreased throughout to the third-year post-exposure before recovering. By the end of the fifth-year post-exposure, the mean HRQoL of the exposed group was still lower than their pre-exposure HRQoL. Model 1 in Table 2 shows results for the DiD analysis for the impact of a serious injury or illness on QALYs. Overall, the findings show that the exposed group had significantly lower QALYs than the unexposed group with a total QALY loss of 0.154 over a six-year period. The year-on-year analysis reveals that, compared to the unexposed group, the exposed group had QALY loss of 0.020 during the year of exposure, which increases to 0.032 by the end of the third-year post-exposure. Although QALY loss starts to recover at 4-year post-exposure, it is still substantial at 0.025 by the end of the fifth-year post-exposure (see Appendix C for the full DiD results).

Additional results on the loss in physical health and mental health after a serious injury or illness are reported in Models 2 and 3 in Table 2. The findings show that the exposed group had greater loss in physical and mental health during the six-year follow-up period compared to the unexposed group. Overall, the exposed group had a 6-year physical health loss of 6.690 units (95%CI: -8.746 to -4.633) compared to the unexposed group. The corresponding 6-year loss in mental health for the exposed group is 3.269 units (95%CI: -4.883 to -1.656). The year-on-year analysis shows that the loss in physical health post-exposure increases to a peak of 7.955 (95%CI: -10.657 to -5.252) by the end of the third year before it starts to recover throughout the fifth-year post-exposure. The mental health loss, on the other hand, reaches its peak during the second-year post-exposure and recovers thereafter, although the rate of recovery is slower than physical health (see Appendices D and E for the full DiD results).

**Table 2:** Difference-in-difference estimates of the recovery of health-related quality of life after a serious injury or illness

	ATT	95% CIs	*p*-value	*N*	*R*-squared
**(Model 1) SF-6D** (QALYs)					
t + 0	-0.020	-0.029 to -0.014	< 0.01	4,572	0.195
t + 1	-0.024	-0.039 to -0.010	< 0.01	4,724	0.201
t + 2	-0.026	-0.041 to -0.012	< 0.01	4,512	0.208
t + 3	-0.032	-0.046 to -0.018	< 0.01	4,592	0.203
t + 4	-0.027	-0.042 to-0.013	< 0.01	4,376	0.199
t + 5	-0.025	-0.040 to -0.010	< 0.01	4,354	0.192
Total	-0.026 (0.154)	-0.037 to -0.015	< 0.01	15,275	0.199
**(Model 2) PH**					
t + 0	-6.013	-8.695 to -3.332	< 0.01	4,857	0.288
t + 1	-5.588	-8.289 to -2.887	< 0.01	4,993	0.288
t + 2	-7.676	-10.383 to -4.969	< 0.01	4,845	0.293
t + 3	-7.955	-10.657 to -5.252	< 0.01	4,862	0.301
t + 4	-5.983	-8.806 to -3.159	< 0.01	4,681	0.282
t + 5	-6.741	-9.531 to -3.951	< 0.01	4,682	0.295
Total	-6.690	-8.746 to -4.633	< 0.01	16,285	0.303
**(Model 3) MH**					
t + 0	-3.207	-5.324 to -1.091	< 0.01	4,840	0.104
t + 1	-2.189	-4.292 to -0.085	< 0.05	4,989	0.103
t + 2	-4.170	-6.317 to -2.022	< 0.01	4,841	0.107
t + 3	-3.657	-5.774 to -1.540	< 0.01	4,905	0.104
t + 4	-3.205	-5.384 to -1.026	< 0.01	4,700	0.116
t + 5	-3.381	-5.544 to -1.219	0.01	4,734	0.103
Total	-3.269	-4.883 to -1.656	< 0.01	16,369	0.101

ATT: average treatment effect on the treated; MH: mental health; PH: physical health; QALYs: quality-adjusted life years. Control variables include exposure status, follow up time, gender, age, marital status, educational status, body mass index (BMI), employment status and remoteness living. Values in parentheses represent total QALY loss over the six years follow up period

### Economic cost of QALY loss due to a serious injury or illness

**Fig. 2:**
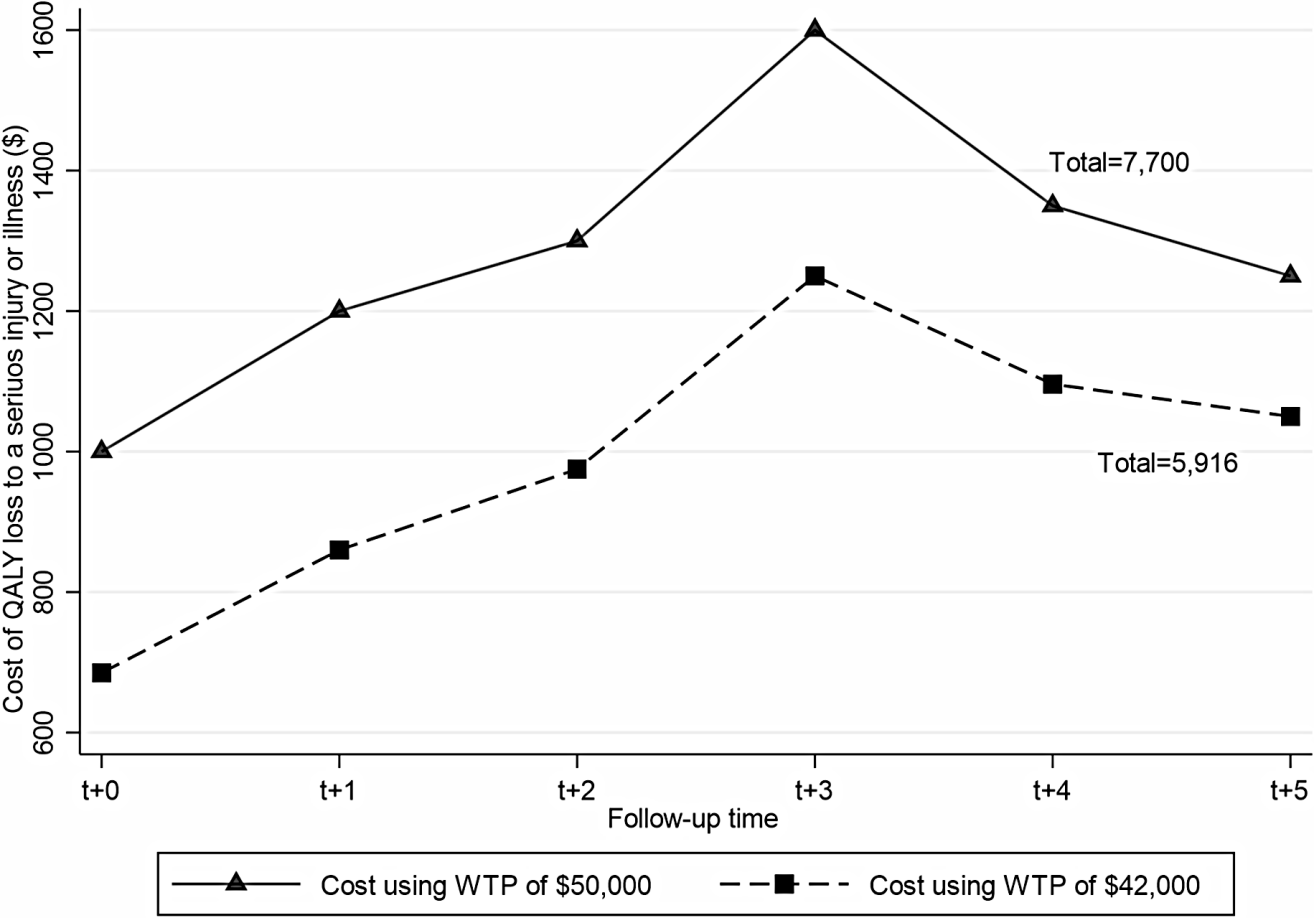
Economic cost of quality-adjusted life years (QALYs) lost due to serious injury or illness

Figure 2 shows the economic cost (intrinsic value) of QALY loss due to a serious injury or illness (with calculation details available in Appendix F). The cost of QALY loss due to serious injury or illness during the year of exposure was $685 per person, which increases to $860 per person at year one post-exposure. Cost continues to increase to a peak of $1,250 during the third year, then decreases to $1,050 by the end of the fifth year. Within six years, serious injury or illness was associated with $5,916 per person lost in QALYs, which translates to a lower bound estimate of $7.2 million for the entire cohort in this study.

### Sensitivity tests

We now perform several sensitivity tests to ensure consistency in our main results. Firstly, to address any contamination effect from follow-up injuries or illnesses, we performed two things: (1) we re-estimated our DiD model and included a dummy variable for whether the individual had any serious injury or illness during the follow-up period together with all the control variables explained earlier. The results in Table 3 show that the decrements in HRQoL over time are qualitatively similar to our baseline estimates, with minimal quantitative variation. (2) we re-estimated the DiD model for only those who did not have any subsequent serious injury or illness during the follow-up period and the results are reported in Table 4. Consistent with our baseline results, we still find a compromised HRQoL during the six-year follow-up period.

**Table 3:** Difference-in-difference estimates of health-related quality of life after a serious injury or illness (controlling for any subsequent serious injury or illness)

	ATT	95% CI	*p*-value	*N*	*R*-squared
(Model 1) SF-6D (QALYs)					
t + 1	-0.020	-0.034 to -0.006	< 0.01	4,724	0.220
t + 2	-0.021	-0.036 to -0.007	< 0.01	4,512	0.224
t + 3	-0.026	-0.041 to -0.012	< 0.01	4,592	0.214
t + 4	-0.022	-0.037 to -0.008	< 0.01	4,376	0.209
t + 5	-0.021	-0.035 to -0.006	< 0.01	4,354	0.203
Total	-0.011 (0.055)	-0.022 to -0.001	< 0.05	15,275	0.216
(Model 2) PH					
t + 1	-4.492	-7.176 to -1.809	< 0.01	4,993	0.302
t + 2	-6.816	-9.521 to -4.111	< 0.01	4,845	0.301
t + 3	-7.112	-9.817 to -4.408	< 0.01	4,862	0.307
t + 4	-5.052	-8.190 to -2.528	< 0.01	4,681	0.286
t + 5	-6.186	-8.978 to -3.395	< 0.01	4,682	0.298
Total	-4.455	-6.733to -2.377	< 0.01	16,285	0.310
(Model 3) MH					
t + 1	-1.622	-3.724 to 0.479	0.130	4,989	0.112
t + 2	-3.672	-5.819 to -1.526	< 0.01	4,841	0.114
t + 3	-3.261	-5.382 to -1.141	< 0.01	4,905	0.107
t + 4	-2.788	-4.967 to -0.608	< 0.05	4,700	0.120
t + 5	-2.772	-4.928 to -0.614	< 0.05	4,734	0.108
Total	-1.656	-3.291 to -0.021	< 0.05	16,369	0.110

ATT: average treatment effect on the treated; MH: mental health; PH: physical health; QALYs: quality-adjusted life years. Control variables include exposure status, follow-up time, any subsequent serious illness, gender, age, marital status, educational status, body mass index (BMI), employment status and remoteness living. Values in parentheses represent total QALY loss over the six years follow up period

**Table 4:** Difference-in-difference estimates of health-related quality of life after a serious injury or illness (excluding those with any subsequent serious injury or illness)

	ATT	95% CI	*p*-value	*n*	*R*-squared
(Model 1) SF-6D (QALYs)					
t + 1	-0.021	-0.037 to -0.005	< 0.05	3,766	0.184
t + 2	-0.018	-0.034 to -0.002	< 0.05	3,595	0.204
t + 3	-0.019	-0.034 to -0.003	< 0.05	3,812	0.196
t + 4	-0.016	-0.032 to 0.0003	< 0.10	3,606	0.191
t + 5	-0.017	-0.033 to -0.0005	< 0.05	3,633	0.191
Total	-0.018 (0.090)	-0.034 to -0.003	< 0.05	6,342	0.170
(Model 2) PH					
t + 1	-4.170	-7.110 to -1.230	< 0.01	4,103	0.278
t + 2	-5.856	-8.832 to -2.887	< 0.01	3,953	0.294
t + 3	-6.117	-9.028 to -3.206	< 0.01	4,114	0.309
t + 4	-4.631	-7.743 to -1.519	< 0.01	3,942	0.278
t + 5	-5.541	-8.644 to -2.437	< 0.01	3,930	0.283
Total	-6.217	-9.084 to -3.349	< 0.01	7,453	0.295
(Model 3) MH					
t + 1	-1.936	-4.244 to 0.371	0.100	4,136	0.100
t + 2	-3.406	-5.760 to -1.053	< 0.01	4,007	0.098
t + 3	-2.454	-4.747 to -1.161	< 0.05	4,176	0.101
t + 4	-2.473	-4.856 to -0.090	< 0.05	3,990	0.113
t + 5	-1.652	-3.986 to 0.682	0.165	4,009	0.104
Total	-2.079	-4.317 to -0.159	< 0.100	7,757	0.090

ATT: average treatment effect on the treated; MH: mental health; PH: physical health; QALYs: quality-adjusted life years Control variables include exposure status, follow up time, gender, age, marital status, educational status, body mass index (BMI), employment status and remoteness living. Values in parentheses represent total QALY loss over the six years follow up period

Next, we test for possible omitted variable bias in our DiD specification. To do this, we respecify Eq. (1) and included an additional set of control variables. Specifically, we include state fixed effects, financial fragility, which is major concern in Australia [28], and social capital as some studies have shown a strong positive association between social capital and quality of life [29]. The results in Table 5 show that our findings are stable across all models even after the inclusion of these additional control variables, suggesting that our estimates are not compromised by omitted variable bias.

**Table 5:** Difference-in-difference estimates of health-related quality of life after a serious injury or illness after including set of additional control variables

	ATT	95% CI	*p*-value	*N*	*R*-squared
(Model 1) SF-6D (QALYs)					
t + 0	-0.020	-0.034 to -0.006	< 0.01	4,572	0.242
t + 1	-0.024	-0.038 to -0.010	< 0.01	4,724	0.243
t + 2	-0.024	-0.038 to -0.010	< 0.01	4,512	0.255
t + 3	-0.029	-0.043 to -0.015	< 0.01	4,592	0.252
t + 4	-0.027	-0.041 to-0.012	< 0.01	4,376	0.247
t + 5	-0.024	-0.038 to -0.010	< 0.01	4,354	0.240
Total	-0.025 [0.150]	-0.035 to -0.015	< 0.01	15,275	0.249
(Model 2) PH					
t + 0	-6.133	-8.779 to -3.486	< 0.01	4,857	0.308
t + 1	-5.708	-8.372 to -3.043	< 0.01	4,993	0.308
t + 2	-7.470	-10.140 to -4.799	< 0.01	4,845	0.313
t + 3	-7.666	-10.324 to -5.007	< 0.01	4,862	0.325
t + 4	-6.019	-8.800 to -3.237	< 0.01	4,681	0.304
t + 5	-6.591	-9.348 to -3.835	< 0.01	4,682	0.314
Total	-6.614	-8.642 to -4.585	< 0.01	16,285	0.323
(Model 3) MH					
t + 0	-3.204	-5.223 to -1.185	< 0.01	4,840	0.187
t + 1	-2.213	-4.234 to -0.193	< 0.05	4,989	0.173
t + 2	-3.803	-5.859 to -1.747	< 0.01	4,841	0.185
t + 3	-3.302	-5.329 to -1.274	< 0.01	4,905	0.182
t + 4	-3.459	-5.544 to -1.373	< 0.01	4,700	0.193
t + 5	-3.006	-5.074 to -0.937	< 0.01	4,734	0.183
Total	-3.129	-4.674 to -1.585	< 0.01	16,369	0.179

ATT: average treatment effect on the treated; MH: mental health; PH: physical health; QALYs: quality-adjusted life years. Control variables include exposure status, follow up time, gender, age, marital status, educational status, body mass index (BMI), employment status and remoteness living, state fixed effect, financial fragility, and social capital. Financial fragility is measured as a dummy variable that is equal to one if the individual could not easily raise an emergency fund of A$4,000 within one week and zero if otherwise. Social capital is captured as a dummy variable that equals one if the individual thinks he/she has a lot of friends and zero if otherwise. Values in parentheses represent total QALY loss over the six years follow up period

Finally, we explore a possible recall bias in our exposure variable as it is likely that individuals may not recall the severity of the injury or illness when asked 12 months after. To do this, we use two main contemporaneous variables that were asked during the year of injury or illness. During the survey, individuals were asked to rate their current health status on a scale of 1 (poor) to 5 (excellent). They were also asked to compare their current health to one year ago (2012). The results in Table 6 show that the exposed group reported about 0.4-folds lower rating of their health during the year of illness compared to the unexposed group. Similarly, the exposed group were about 0.30 percentage points more likely to report that their current health is worse than the previous year compared to the unexposed group. These two contemporaneous findings suggest that recall bias does not compromise the reliability of our estimates.

**Table 6:** Contemporaneous association between serious illness and self-assessed health

	Current health status	Worse health than last year
Serious injury or illness	-0.384*** (-0.461, -0.306)	0.270*** (0.233, 0.307)
Control variables	Yes	Yes
R-squared	0.192	0.122
N	2,193	2,193

95% confidence intervals in parentheses, *** *p* < 0.01, Control variables include exposure status, follow-up time, gender, age, marital status, educational status, body mass index (BMI), employment status and remoteness living. The ordinary least square regression was used to estimate the association between exposure to a serious injury or illness and contemporaneous health status while the linear probability model was used to examine how the exposed and unexposed groups compared their current health to a year prior. These analyses were done on the matched cohort and all control variables discussed in the baseline DiD regression were included in the models

## Discussion

Serious injuries or illnesses can have severe consequences on an individual’s HRQoL. This study has utilised longitudinal cohort data of individuals who reported having had any serious injury or illness to explore their recovery trajectory over a six-year period, focusing on their HRQoL. A PSM-DiD approach was used to estimate the recovery of HRQoL after a serious injury or illness.

Our findings are three-fold. Firstly, the QALYs lost to serious injury or illness reaches its trough at year three post-exposure, thereafter, it begins to recover throughout year five post-exposure. A previous study in Australia found that those exposed to any serious injury or illness had 0.059 lower health utility scores than those who were unexposed [10]. While this estimate shows the health utilities lost, there are no details on the time dimension for these health utilities lost as well as when and whether the utilities lost were recovered. Our finding that the annual QALY loss during the six-year follow-up period varied between 0.020 and 0.032 is consistent with previous finding from the United States which showed that QALY loss in the first-year post-injury is within 0.005 to 0.109 [16]. Hence, our year-on-year estimates of QALYs lost provide useful information for economic evaluation of health interventions that seek to improve the recovery of those who have suffered any serious injury or illness. Particularly, the finding that QALYs lost begin to recover after the third-year post-exposure highlights the need for critical intervention during the first three years of serious injury or illness. That is, while designing interventions to target the entire recovery journey of the affected individuals can be costly to the health system, interventions that target the first three years of serious injury or illness may be a cost-effective option.

Secondly, our study has shown that although the loss in physical health is greater than mental health, the recovery in mental health is slower than that of physical health as overall HRQoL begins to recover. While previous studies have shown that serious injuries or illnesses are associated with poor physical and mental health, they have had shorter follow-up periods [30, 31]. However, evidence from the psychiatry literature suggests that the mental distress after a traumatic event can stay longer and develop into post-traumatic stress disorder (PTSD), which often kicks in after six months post-exposure [32]. PTSD is costly to the affected individuals as they have negative emotions, thoughts and memories that interfere with their daily lives. Therefore, our finding that those exposed to serious injuries or illnesses have about three units lower mental health scores than the unexposed group, even after five years post-exposure, provides critical evidence for the design of long-lasting policies to support people who are recovering from a traumatic health event.

Finally, our study has estimated that the intrinsic value (economic cost) of QALYs lost to a serious injury or illness is substantial, increasing from $685 per person during the year of event to a peak of $1,250 per person by the end of the third-year post-event. Over a six-year period, the cost of QALY loss translates to $5,916, equivalent to a lower bound cost of $7.2 million for the entire study cohort. The burden of personal injuries or illnesses includes healthcare costs, lost productivity, presenteeism, and compromised HRQoL. With the rise of digital transformation in Australia, underpinned by the National Digital Health Strategy [33], estimation of healthcare costs associated with a particular injury or illness has been easy through access to hospital data, Medicare Benefits Schedule and Pharmaceutical Benefit Scheme datasets. These data are mostly useful for cost analysis from the perspective of the healthcare funders as the datasets often do not include measures for patient-reported outcomes that will enable proper analysis of the overall health burden from the patient’s perspective. Previous studies have also quantified lost productivity and earnings due to serious illnesses [11–13], with the cost of QALYs lost underexplored. Our cost analysis of the HILDA dataset provides opportunities to explore those other indirect costs attributable to serious injuries or illnesses. For example, using our cost estimates and the number of hospital admissions due to serious injuries alone (548,654 hospitalisations) in 2022-23 as reported by the Australia Institute of Health and Welfare [34], the cost of compromised HRQoL for the patients during the year of exposure amounted to about $379 million. By the end of the third-year post-exposure, it will cost about $2.1 billion to the affected individuals due to loss in HRQoL. These provocative findings highlight the need to design appropriate support packages for those who suffer any serious injury of illness. Such support packages may include bulk billing some specialist and allied health services that can directly improve the recovery of people’s HRQoL after a serious injury or illness. Whatever combination of support services should be valued between $685 to $1,250 per person per annum, as our findings show that this is how much society is willing to pay to compensate for the lost HRQoL. That said, such interventions will require proper economic evaluation to determine their cost-effectiveness and sustainability.

Despite the significant contributions our study makes to the literature, it has some limitations that need to be highlighted. Firstly, our exposure variable was captured to include both injury and illness events, making it difficult to disentangle the independent effects of injuries from other illnesses. Hence, using our QALY estimates for economic evaluation should be done with caution. That said, since our HRQoL measure (SF-36) is not disease-specific but a generic constructs, it best captures one’s HRQoL irrespective of the type of health event. Secondly, although our exposure variable is intended to capture a serious injury or illness that occurred in the last twelve months preceding the survey year, it is possible that some injuries or illnesses occurred during the survey year. This can potentially bias our findings depending on the proportion of participants who had their injury or illness in the survey year. Finally, it is possible that some of the individuals in the exposure group experiencing very severe injury or illness may drop out of the sample during the follow up periods. This partly explains our relatively smaller QALY estimates, hence our findings should be interpreted as lower bound.

In conclusion, this study has utilised a unique dataset and methodology to examine the recovery of HRQoL after a serious injury or illness. The key finding is that QALY loss begins to recover after year three post-exposure. However, the mental health losses permeate into the longer term. Follow-up care designed to mitigate the impacts of a serious illness on people’s HRQoL should consider cost-effective strategies that are long-lasting and support those affected throughout, at least, the first three years of their serious injury or illness.

## Electronic supplementary material

Below is the link to the electronic supplementary material.

Supplementary Material 1

Supplementary Material 2

## Data Availability

The HILDA data are available through the University of Melbourne Faculty of Business and Economics at https://www.melbourneinstitute.com/hilda/.
