# Supplementary material for: Recovery trajectories after a serious injury or illness: a longitudinal evaluation of health-related quality of life in an Australian cohort
Source: Qual Life Res. 2025 Feb 17;34(6):1657–67. doi: 10.1007/s11136-025-03919-w (PMC12119750; doi:10.1007/s11136-025-03919-w)
Supplement: Supplementary file 1 — Supplementary Material 1 [file 11136_2025_3919_MOESM1_ESM.docx]

Appendices

Appendix A: Cohort identification and follow-ups

|  |  |  | Before PSM | |  | After PSM | |  |
| --- | --- | --- | --- | --- | --- | --- | --- | --- |
| Year | Time | Definition | Exposed | Control | | Exposed | Control | |
| 2012 | t-1 | Pre-exposure (baseline) | 1,212 | 12,203 | | 1,212 | 1,216 | |
| 2013 | t+0 | Year of exposure | 1,202 | 12,686 | | 1,067 | 1,125 | |
| 2014 | t+1 | 1-year post-exposure (cohort identification) | 1,428 | 13,501 | | 1,175 | 1,152 | |
| 2015 | t+2 | 2-years post-exposure | 1,316 | 12,821 | | 1,104 | 1,045 | |
| 2016 | t+3 | 3-years post-exposure | 1,398 | 13,052 | | 1,143 | 1,039 | |
| 2017 | t+4 | 4-years post-exposure | 1,134 | 12,873 | | 931 | 1,067 | |
| 2018 | t+5 | 5-years post-exposure | 1,252 | 12,226 | | 1,006 | 94 | |
| Total |  |  | 8,942 | 89,362 | | 7,638 | 7,638 | |

Note: Not all individuals (both in the exposed and control groups) had equal follow up period. PSM: propensity score matching.

Appendix B: Long-term health-related quality of life after a serious injury or illness

| Time | SF-6D  Mean (SD) | PH  Mean (SD) | MH  Mean (SD) |
| --- | --- | --- | --- |
| t-1 (Baseline) | 0.699 (0.134) | 72.670 (27.422) | 70.581(19.497) |
| t+0 (Year of exposure) | 0.686 (0.137) | 70.483 (27.952) | 69.873 (19.373) |
| t+1(1-year post-exposure) | 0.678 (0.137) | 68.904 (29.163) | 69.354 (19.832) |
| t+2 (2-years post-exposure) | 0.674(0.136) | 68.715 (28.691) | 69.025(20.114) |
| t+3 (3-years post-exposure) | 0.680 (0.141) | 68.468 (29.106) | 69.822 (19.939) |
| t+4 (4-years post-exposure) | 0.681 (0.138) | 69.051 (29.080) | 70.211 (19.829) |
| t+5 (5-years post-exposure) | 0.682 (0.134) | 67.570 (29.230) | 69.117 (19.561) |

Note: SD is standard deviation

Appendix C: Difference-in-difference estimates of the effect of a serious illness on SF-6D utilities

| VARIABLES | t+0 | t+1 | t+2 | t+3 | t+4 | t+5 | Total |
| --- | --- | --- | --- | --- | --- | --- | --- |
| Exposed*Post | -0.020*** | -0.024*** | -0.026*** | -0.032*** | -0.027*** | -0.025*** | -0.026*** |
|  | (-0.035 - -0.006) | (-0.039 - -0.010) | (-0.041 - -0.012) | (-0.046 - -0.018) | (-0.042 - -0.013) | (-0.040 - -0.010) | (-0.037 - -0.015) |
| Exposed | -0.041*** | -0.041*** | -0.041*** | -0.041*** | -0.041*** | -0.041*** | -0.041*** |
|  | (-0.051 - -0.031) | (-0.051 - -0.031) | (-0.050 - -0.031) | (-0.051 - -0.031) | (-0.051 - -0.031) | (-0.051 - -0.031) | (-0.051 - -0.031) |
| Post | 0.013*** | 0.013** | 0.012** | 0.017*** | 0.019*** | 0.010* | 0.014*** |
|  | (0.003 - 0.023) | (0.003 - 0.023) | (0.001 - 0.022) | (0.007 - 0.027) | (0.009 - 0.029) | (-0.001 - 0.021) | (0.006 - 0.022) |
| female | -0.021*** | -0.019*** | -0.021*** | -0.019*** | -0.021*** | -0.022*** | -0.019*** |
|  | (-0.029 - -0.014) | (-0.027 - -0.012) | (-0.028 - -0.013) | (-0.026 - -0.011) | (-0.029 - -0.014) | (-0.030 - -0.014) | (-0.023 - -0.015) |
| Age groups (ref= 15-24) |  |  |  |  |  |  |  |
| 25-44 | -0.044*** | -0.044*** | -0.043*** | -0.035*** | -0.052*** | -0.048*** | -0.036*** |
|  | (-0.060 - -0.028) | (-0.060 - -0.028) | (-0.059 - -0.026) | (-0.052 - -0.019) | (-0.069 - -0.034) | (-0.066 - -0.029) | (-0.046 - -0.026) |
| 45-64 | -0.059*** | -0.056*** | -0.052*** | -0.048*** | -0.063*** | -0.055*** | -0.050*** |
|  | (-0.075 - -0.042) | (-0.072 - -0.039) | (-0.068 - -0.035) | (-0.065 - -0.030) | (-0.080 - -0.045) | (-0.074 - -0.037) | (-0.060 - -0.040) |
| ≥65 | -0.026*** | -0.023** | -0.019** | -0.016 | -0.025** | -0.025** | -0.019*** |
|  | (-0.044 - -0.007) | (-0.041 - -0.005) | (-0.038 - -0.001) | (-0.035 - 0.003) | (-0.044 - -0.005) | (-0.045 - -0.004) | (-0.030 - -0.008) |
| Marital status (ref=married) |  |  |  |  |  |  |  |
| De facto | -0.004 | -0.002 | -0.007 | -0.005 | -0.003 | -0.009 | -0.003 |
|  | (-0.017 - 0.008) | (-0.015 - 0.010) | (-0.019 - 0.005) | (-0.018 - 0.007) | (-0.015 - 0.010) | (-0.021 - 0.004) | (-0.010 - 0.004) |
| Separated | -0.050*** | -0.039*** | -0.039*** | -0.038*** | -0.057*** | -0.057*** | -0.045*** |
|  | (-0.071 - -0.030) | (-0.059 - -0.019) | (-0.060 - -0.018) | (-0.059 - -0.017) | (-0.077 - -0.036) | (-0.078 - -0.036) | (-0.056 - -0.034) |
| Divorced | -0.047*** | -0.046*** | -0.051*** | -0.044*** | -0.042*** | -0.045*** | -0.043*** |
|  | (-0.060 - -0.034) | (-0.058 - -0.034) | (-0.063 - -0.038) | (-0.057 - -0.032) | (-0.054 - -0.029) | (-0.059 - -0.032) | (-0.050 - -0.037) |
| Widowed | -0.012* | -0.020*** | -0.023*** | -0.031*** | -0.022*** | -0.022*** | -0.023*** |
|  | (-0.026 - 0.002) | (-0.034 - -0.006) | (-0.037 - -0.009) | (-0.045 - -0.017) | (-0.037 - -0.008) | (-0.036 - -0.008) | (-0.031 - -0.016) |
| Never married | -0.008 | -0.005 | -0.009 | -0.011* | -0.014** | -0.012* | -0.014*** |
|  | (-0.020 - 0.005) | (-0.018 - 0.007) | (-0.022 - 0.004) | (-0.023 - 0.002) | (-0.026 - -0.001) | (-0.024 - 0.000) | (-0.020 - -0.007) |
| Education (ref=≤ year 12) |  |  |  |  |  |  |  |
| Diploma | 0.008* | 0.011*** | 0.004 | 0.015*** | 0.016*** | 0.012*** | 0.010*** |
|  | (-0.001 - 0.016) | (0.003 - 0.020) | (-0.004 - 0.013) | (0.006 - 0.023) | (0.007 - 0.024) | (0.004 - 0.021) | (0.005 - 0.015) |
| ≥Bachelor’s | 0.021*** | 0.025*** | 0.019*** | 0.022*** | 0.026*** | 0.025*** | 0.023*** |
|  | (0.011 - 0.031) | (0.016 - 0.035) | (0.009 - 0.029) | (0.012 - 0.032) | (0.015 - 0.036) | (0.015 - 0.035) | (0.017 - 0.028) |
| LF status (ref=employed) |  |  |  |  |  |  |  |
| Unemployed | -0.050*** | -0.058*** | -0.059*** | -0.061*** | -0.052*** | -0.044*** | -0.051*** |
|  | (-0.071 - -0.029) | (-0.078 - -0.038) | (-0.081 - -0.038) | (-0.083 - -0.040) | (-0.075 - -0.030) | (-0.067 - -0.021) | (-0.063 - -0.039) |
| Not in LF | -0.093*** | -0.095*** | -0.098*** | -0.092*** | -0.093*** | -0.089*** | -0.089*** |
|  | (-0.102 - -0.084) | (-0.104 - -0.086) | (-0.107 - -0.089) | (-0.101 - -0.083) | (-0.102 - -0.083) | (-0.099 - -0.080) | (-0.094 - -0.084) |
| Usual residence (ref=major city) |  |  |  |  |  |  |  |
| Regional | -0.003 | 0.001 | 0.001 | -0.003 | -0.003 | -0.003 | -0.002 |
|  | (-0.011 - 0.004) | (-0.007 - 0.008) | (-0.007 - 0.008) | (-0.011 - 0.005) | (-0.011 - 0.005) | (-0.011 - 0.005) | (-0.006 - 0.003) |
| Remote | 0.033** | 0.022 | 0.032** | 0.023* | 0.024 | 0.019 | 0.012 |
|  | (0.002 - 0.063) | (-0.009 - 0.052) | (0.003 - 0.062) | (-0.003 - 0.050) | (-0.009 - 0.057) | (-0.017 - 0.054) | (-0.004 - 0.029) |
| BMI | -0.001*** | -0.001*** | -0.001*** | -0.001*** | -0.001*** | -0.001*** | -0.001*** |
|  | (-0.001 - -0.000) | (-0.001 - -0.000) | (-0.001 - -0.001) | (-0.001 - -0.001) | (-0.001 - -0.000) | (-0.001 - -0.001) | (-0.001 - -0.001) |
| Constant | 0.817*** | 0.813*** | 0.824*** | 0.815*** | 0.823*** | 0.822*** | 0.811*** |
|  | (0.796 - 0.838) | (0.793 - 0.833) | (0.803 - 0.845) | (0.793 - 0.836) | (0.801 - 0.844) | (0.799 - 0.844) | (0.798 - 0.824) |
|  |  |  |  |  |  |  |  |
| Observations | 4,572 | 4,724 | 4,512 | 4,592 | 4,376 | 4,354 | 15,275 |
| R-squared | 0.195 | 0.201 | 0.208 | 0.203 | 0.199 | 0.192 | 0.199 |

Robust ci in parentheses, *** p<0.01, ** p<0.05, * p<0. LF =Labour Force. BMI= Body Mass Index

Appendix D: Difference-in-difference estimates of the effect of a serious illness on physical health

| VARIABLES | t+0 | t+1 | t+2 | t+3 | t+4 | t+5 | Total |
| --- | --- | --- | --- | --- | --- | --- | --- |
| Exposed*Post | -6.013*** | -5.588*** | -7.676*** | -7.955*** | -5.983*** | -6.741*** | -6.690*** |
|  | (-8.695 - -3.332) | (-8.289 - -2.887) | (-10.383 - -4.969) | (-10.657 - -5.252) | (-8.806 - -3.159) | (-9.531 - -3.951) | (-8.746 - -4.633) |
| Exposed | -1.763* | -1.844* | -1.727* | -1.804* | -1.795* | -1.719* | -1.800* |
|  | (-3.655 - 0.128) | (-3.738 - 0.049) | (-3.622 - 0.167) | (-3.701 - 0.093) | (-3.689 - 0.100) | (-3.613 - 0.175) | (-3.694 - 0.095) |
| Post | 4.856*** | 3.846*** | 5.479*** | 5.549*** | 4.728*** | 5.082*** | 4.990*** |
|  | (2.993 - 6.719) | (1.963 - 5.730) | (3.604 - 7.354) | (3.679 - 7.419) | (2.812 - 6.644) | (3.137 - 7.028) | (3.536 - 6.443) |
| female | -3.732*** | -3.443*** | -3.137*** | -3.611*** | -3.704*** | -3.378*** | -3.665*** |
|  | (-5.125 - -2.340) | (-4.848 - -2.037) | (-4.554 - -1.720) | (-5.023 - -2.199) | (-5.163 - -2.245) | (-4.831 - -1.925) | (-4.437 - -2.894) |
| Age groups (ref= 15-24) |  |  |  |  |  |  |  |
| 25-44 | -5.009*** | -6.643*** | -6.230*** | -5.044*** | -6.557*** | -7.413*** | -6.469*** |
|  | (-7.709 - -2.310) | (-9.284 - -4.001) | (-9.034 - -3.427) | (-7.987 - -2.101) | (-9.366 - -3.749) | (-10.330 - -4.496) | (-8.109 - -4.829) |
| 45-64 | -16.017*** | -16.353*** | -15.644*** | -15.107*** | -16.120*** | -17.173*** | -15.601*** |
|  | (-18.852 - -13.181) | (-19.180 - -13.525) | (-18.553 - -12.736) | (-18.150 - -12.065) | (-19.035 - -13.205) | (-20.200 - -14.146) | (-17.288 - -13.914) |
| ≥65 | -18.482*** | -20.279*** | -19.171*** | -19.874*** | -19.351*** | -19.948*** | -20.842*** |
|  | (-21.706 - -15.258) | (-23.495 - -17.062) | (-22.457 - -15.885) | (-23.282 - -16.466) | (-22.675 - -16.027) | (-23.373 - -16.522) | (-22.737 - -18.947) |
| Marital status (ref=married) |  |  |  |  |  |  |  |
| De facto | 1.048 | 0.646 | -0.240 | 1.146 | 1.197 | 0.702 | 0.496 |
|  | (-1.020 - 3.116) | (-1.515 - 2.808) | (-2.427 - 1.947) | (-0.997 - 3.289) | (-0.997 - 3.392) | (-1.533 - 2.936) | (-0.673 - 1.665) |
| Separated | -1.108 | -4.573** | -2.838 | -2.208 | -4.621* | -4.148* | -4.005*** |
|  | (-5.391 - 3.174) | (-8.823 - -0.322) | (-7.047 - 1.372) | (-6.586 - 2.171) | (-9.277 - 0.034) | (-8.741 - 0.444) | (-6.236 - -1.773) |
| Divorced | -6.839*** | -6.660*** | -7.863*** | -6.725*** | -6.414*** | -6.405*** | -5.989*** |
|  | (-9.529 - -4.148) | (-9.263 - -4.058) | (-10.485 - -5.241) | (-9.274 - -4.177) | (-9.172 - -3.656) | (-9.043 - -3.766) | (-7.391 - -4.587) |
| Widowed | -6.467*** | -5.769*** | -8.597*** | -9.097*** | -6.982*** | -8.053*** | -8.985*** |
|  | (-9.435 - -3.499) | (-8.793 - -2.746) | (-11.587 - -5.606) | (-12.135 - -6.059) | (-10.049 - -3.915) | (-10.981 - -5.124) | (-10.613 - -7.356) |
| Never married | 0.129 | 0.749 | -0.235 | -0.199 | -0.254 | -0.854 | -0.635 |
|  | (-2.121 - 2.379) | (-1.484 - 2.981) | (-2.445 - 1.975) | (-2.445 - 2.047) | (-2.461 - 1.953) | (-3.078 - 1.369) | (-1.887 - 0.618) |
| Education (ref=≤ year 12) |  |  |  |  |  |  |  |
| Diploma | 2.982*** | 3.772*** | 3.735*** | 4.096*** | 3.574*** | 4.545*** | 3.822*** |
|  | (1.351 - 4.612) | (2.138 - 5.406) | (2.077 - 5.393) | (2.444 - 5.748) | (1.878 - 5.270) | (2.841 - 6.249) | (2.926 - 4.718) |
| ≥Bachelor’s | 7.303*** | 8.418*** | 7.967*** | 8.556*** | 8.286*** | 9.711*** | 8.165*** |
|  | (5.457 - 9.148) | (6.558 - 10.278) | (6.122 - 9.812) | (6.716 - 10.395) | (6.392 - 10.179) | (7.843 - 11.580) | (7.162 - 9.167) |
| LF status (ref=employed) |  |  |  |  |  |  |  |
| Unemployed | -6.876*** | -6.036*** | -5.762*** | -5.542*** | -9.274*** | -6.626*** | -6.356*** |
|  | (-10.600 - -3.152) | (-9.815 - -2.256) | (-9.330 - -2.195) | (-9.230 - -1.855) | (-13.629 - -4.919) | (-10.580 - -2.671) | (-8.529 - -4.183) |
| Not in LF | -17.323*** | -17.152*** | -17.719*** | -16.287*** | -17.863*** | -17.867*** | -16.849*** |
|  | (-19.101 - -15.545) | (-18.957 - -15.346) | (-19.509 - -15.930) | (-18.090 - -14.484) | (-19.728 - -15.998) | (-19.728 - -16.007) | (-17.845 - -15.854) |
| Usual residence (ref=major city) |  |  |  |  |  |  |  |
| Regional | -1.030 | -1.267* | -0.233 | -1.130 | -1.328* | -1.306* | -0.873** |
|  | (-2.490 - 0.430) | (-2.714 - 0.180) | (-1.699 - 1.233) | (-2.587 - 0.328) | (-2.828 - 0.171) | (-2.796 - 0.184) | (-1.671 - -0.075) |
| Remote | -0.778 | -0.764 | 0.756 | -3.129 | -0.553 | 0.017 | -0.249 |
|  | (-6.565 - 5.009) | (-6.851 - 5.324) | (-5.134 - 6.647) | (-8.670 - 2.411) | (-6.642 - 5.536) | (-5.868 - 5.903) | (-3.725 - 3.227) |
| BMI | -0.234*** | -0.203*** | -0.220*** | -0.278*** | -0.181*** | -0.266*** | -0.245*** |
|  | (-0.325 - -0.143) | (-0.290 - -0.116) | (-0.305 - -0.135) | (-0.363 - -0.194) | (-0.272 - -0.089) | (-0.356 - -0.176) | (-0.293 - -0.197) |
| Constant | 97.200*** | 96.725*** | 96.819*** | 97.554*** | 96.638*** | 98.921*** | 97.963*** |
|  | (93.226 - 101.174) | (92.930 - 100.520) | (92.990 - 100.648) | (93.523 - 101.585) | (92.677 - 100.599) | (94.954 - 102.889) | (95.516 - 100.410) |
|  |  |  |  |  |  |  |  |
| Observations | 4,857 | 4,993 | 4,845 | 4,862 | 4,681 | 4,682 | 16,285 |
| R-squared | 0.288 | 0.288 | 0.293 | 0.301 | 0.282 | 0.295 | 0.303 |

Robust ci in parentheses, *** p<0.01, ** p<0.05, * p<0. LF =Labour Force. BMI= Body Mass Index

Appendix E: Difference-in-difference estimates of the effect of a serious illness on mental health

| VARIABLES | t+0 | t+1 | t+2 | t+3 | t+4 | t+5 | Total |
| --- | --- | --- | --- | --- | --- | --- | --- |
| Exposed*Post | -3.207*** | -2.189** | -4.170*** | -3.657*** | -3.205*** | -3.381*** | -3.269*** |
|  | (-5.324 - -1.091) | (-4.292 - -0.085) | (-6.317 - -2.022) | (-5.774 - -1.540) | (-5.384 - -1.026) | (-5.544 - -1.219) | (-4.883 - -1.656) |
| Exposed | -1.555** | -1.554** | -1.538** | -1.556** | -1.519** | -1.548** | -1.660** |
|  | (-3.051 - -0.058) | (-3.051 - -0.057) | (-3.035 - -0.041) | (-3.053 - -0.059) | (-3.018 - -0.021) | (-3.045 - -0.050) | (-3.151 - -0.170) |
| Post | 2.864*** | 1.446* | 1.972*** | 2.176*** | 1.970*** | 1.616** | 1.992*** |
|  | (1.425 - 4.302) | (-0.001 - 2.894) | (0.492 - 3.452) | (0.714 - 3.638) | (0.522 - 3.418) | (0.136 - 3.097) | (0.873 - 3.111) |
| female | -1.667*** | -1.329** | -2.009*** | -1.876*** | -1.853*** | -2.130*** | -1.561*** |
|  | (-2.777 - -0.558) | (-2.444 - -0.213) | (-3.130 - -0.888) | (-2.978 - -0.773) | (-2.971 - -0.735) | (-3.255 - -1.005) | (-2.160 - -0.961) |
| Age groups (ref= 15-24) |  |  |  |  |  |  |  |
| 25-44 | -3.936*** | -4.331*** | -4.509*** | -3.036** | -4.244*** | -4.326*** | -4.435*** |
|  | (-6.285 - -1.586) | (-6.642 - -2.021) | (-6.916 - -2.103) | (-5.447 - -0.625) | (-6.774 - -1.715) | (-6.903 - -1.750) | (-5.874 - -2.997) |
| 45-64 | -3.120** | -3.035** | -2.479* | -1.309 | -3.105** | -2.637* | -2.598*** |
|  | (-5.554 - -0.686) | (-5.474 - -0.595) | (-4.972 - 0.014) | (-3.794 - 1.177) | (-5.733 - -0.477) | (-5.295 - 0.020) | (-4.065 - -1.132) |
| ≥65 | 7.802*** | 7.693*** | 8.541*** | 9.318*** | 8.172*** | 8.091*** | 6.997*** |
|  | (5.142 - 10.463) | (5.045 - 10.341) | (5.817 - 11.264) | (6.583 - 12.053) | (5.303 - 11.040) | (5.165 - 11.017) | (5.409 - 8.586) |
| Marital status (ref=married) |  |  |  |  |  |  |  |
| De facto | -2.444*** | -2.593*** | -2.037** | -1.824** | -3.026*** | -2.422*** | -2.909*** |
|  | (-4.290 - -0.598) | (-4.405 - -0.782) | (-3.875 - -0.199) | (-3.612 - -0.036) | (-4.913 - -1.139) | (-4.228 - -0.616) | (-3.895 - -1.923) |
| Separated | -7.995*** | -6.126*** | -6.002*** | -4.941*** | -7.913*** | -4.666*** | -6.759*** |
|  | (-11.312 - -4.678) | (-9.687 - -2.565) | (-9.634 - -2.370) | (-8.633 - -1.249) | (-11.674 - -4.152) | (-8.117 - -1.214) | (-8.619 - -4.899) |
| Divorced | -6.858*** | -6.097*** | -5.226*** | -5.510*** | -5.748*** | -5.106*** | -5.041*** |
|  | (-8.956 - -4.759) | (-8.206 - -3.988) | (-7.335 - -3.118) | (-7.613 - -3.407) | (-7.816 - -3.679) | (-7.206 - -3.007) | (-6.137 - -3.946) |
| Widowed | -1.193 | 0.214 | -1.862* | -0.727 | -0.769 | -1.220 | 0.322 |
|  | (-3.305 - 0.919) | (-1.903 - 2.332) | (-4.065 - 0.341) | (-2.864 - 1.409) | (-3.018 - 1.481) | (-3.297 - 0.858) | (-0.811 - 1.455) |
| Never married | -3.226*** | -3.428*** | -3.490*** | -3.081*** | -4.892*** | -3.517*** | -4.797*** |
|  | (-5.184 - -1.269) | (-5.307 - -1.548) | (-5.404 - -1.576) | (-4.946 - -1.216) | (-6.757 - -3.027) | (-5.390 - -1.645) | (-5.858 - -3.736) |
| Education (ref=≤ year 12) |  |  |  |  |  |  |  |
| Diploma | 2.125*** | 1.827*** | 2.065*** | 2.278*** | 2.724*** | 2.219*** | 1.862*** |
|  | (0.857 - 3.392) | (0.578 - 3.076) | (0.786 - 3.344) | (1.012 - 3.545) | (1.440 - 4.008) | (0.925 - 3.514) | (1.172 - 2.552) |
| ≥Bachelor’s | 2.708*** | 3.080*** | 3.157*** | 3.011*** | 3.943*** | 3.352*** | 2.925*** |
|  | (1.236 - 4.179) | (1.609 - 4.550) | (1.664 - 4.649) | (1.540 - 4.481) | (2.458 - 5.428) | (1.880 - 4.824) | (2.150 - 3.699) |
| LF status (ref=employed) |  |  |  |  |  |  |  |
| Unemployed | -8.494*** | -8.661*** | -7.531*** | -9.526*** | -9.416*** | -8.286*** | -8.118*** |
|  | (-11.925 - -5.063) | (-11.946 - -5.375) | (-10.743 - -4.318) | (-13.034 - -6.017) | (-13.009 - -5.823) | (-11.943 - -4.629) | (-10.016 - -6.221) |
| Not in LF | -9.326*** | -10.555*** | -9.978*** | -10.060*** | -10.138*** | -9.885*** | -9.037*** |
|  | (-10.788 - -7.864) | (-11.982 - -9.128) | (-11.430 - -8.526) | (-11.506 - -8.614) | (-11.645 - -8.631) | (-11.376 - -8.393) | (-9.833 - -8.242) |
| Usual residence (ref=major city) |  |  |  |  |  |  |  |
| Regional | 0.606 | 0.627 | 0.825 | 0.697 | 0.931 | 0.536 | 0.765** |
|  | (-0.547 - 1.759) | (-0.509 - 1.763) | (-0.332 - 1.981) | (-0.450 - 1.844) | (-0.226 - 2.088) | (-0.624 - 1.696) | (0.146 - 1.384) |
| Remote | 5.369*** | 2.908 | 2.936 | 4.427** | 5.197** | 4.979*** | 4.234*** |
|  | (2.011 - 8.726) | (-0.959 - 6.776) | (-1.313 - 7.186) | (0.772 - 8.083) | (1.145 - 9.248) | (1.191 - 8.766) | (2.095 - 6.373) |
| BMI | -0.011 | 0.007 | 0.008 | -0.032 | 0.007 | -0.021 | -0.004 |
|  | (-0.077 - 0.054) | (-0.058 - 0.072) | (-0.056 - 0.072) | (-0.096 - 0.032) | (-0.060 - 0.073) | (-0.089 - 0.047) | (-0.040 - 0.031) |
| Constant | 75.194*** | 75.101*** | 74.570*** | 74.456*** | 74.851*** | 75.457*** | 75.098*** |
|  | (72.144 - 78.245) | (72.100 - 78.103) | (71.531 - 77.609) | (71.384 - 77.528) | (71.719 - 77.983) | (72.291 - 78.622) | (73.144 - 77.051) |
|  |  |  |  |  |  |  |  |
| Observations | 4,840 | 4,989 | 4,841 | 4,905 | 4,700 | 4,734 | 16,369 |
| R-squared | 0.104 | 0.103 | 0.107 | 0.104 | 0.116 | 0.103 | 0.101 |

Robust ci in parentheses, *** p<0.01, ** p<0.05, * p<0. LF =Labour Force. BMI= Body Mass Index

Appendix F: Economic cost of QALYs lost to a serious illness

| Time | QALY loss | Cost of QALY loss (A$) |
| --- | --- | --- |
| Using WTP per QALY of $42,000 (adjusted for inflation) |  |  |
| t+0 | 0.020 | 685 (95% CI:479 to 993) |
| t+1 | 0.024 | 860 (95% CI:358 to 1,398) |
| t+2 | 0.026 | 975 (95% CI:450 to 1,538) |
| t+3 | 0.032 | 1,250 (95% CI:703 to 1,798) |
| t+4 | 0.027 | 1,096 (95% CI:528 to 1,705) |
| t+5 | 0.025 | 1,050 (95% CI:420 to 1,680) |
| Total | 0.156 | 5,916 (95% CI:2,939 to 9,110) |
| Using WTP for health intervention of $50,000 (unadjusted for inflation) |  |  |
| t+0 | 0.020 | 1,000 (95% CI:700 to 1,450) |
| t+1 | 0.024 | 1,200 (95% CI:500 to 1,950) |
| t+2 | 0.026 | 1,300 (95% CI:600 to 2,050) |
| t+3 | 0.032 | 1,600 (95% CI:900 to 2,300) |
| t+4 | 0.027 | 1,350 (95% CI:650 to 2,100) |
| t+5 | 0.025 | 1,250 (95% CI:500 to 2,000) |
| Total | 0.156 | 7,700 (95% CI:4500 to 11,100) |

Note: WTP is Willingness to pay.
