# Supplementary material for: Recovery trajectories after a serious injury or illness: a longitudinal evaluation of health-related quality of life in an Australian cohort
Source: Qual Life Res. 2025 Feb 17;34(6):1657–67. doi: 10.1007/s11136-025-03919-w (PMC12119750; doi:10.1007/s11136-025-03919-w)
Supplement: Supplementary file 2 — Supplementary Material 2 [file 11136_2025_3919_MOESM2_ESM.docx]

STROBE Statement—checklist of items that should be included in reports of observational studies

**Recovery trajectories after a serious injury or illness: a longitudinal evaluation of health-related quality of life in an Australian cohort**

|  | Item No | Recommendation | Page  No |
| --- | --- | --- | --- |
| **Title and abstract** | 1 | (*a*) Indicate the study’s design with a commonly used term in the title or the abstract | This is indicated in the abstract on the title page (i.e., page 1) |
|  |  | (*b*) Provide in the abstract an informative and balanced summary of what was done and what was found | The study’s methods used, and findings are in the abstract on the title page (i.e., page 1) |
| Introduction | | | |
| Background/rationale | 2 | Explain the scientific background and rationale for the investigation being reported | Page 2, paragraphs 3  *This paragraph provides the study's rationale, including the research gap.* |
| Objectives | 3 | State specific objectives, including any prespecified hypotheses | Page 3, paragraph 1  *The study’s objective can be found in this paragraph.* |
| Methods | | | |
| Study design | 4 | Present key elements of study design early in the paper | Page 3, paragraph 1  *This paragraph indicates that we apply a quantitative research design and statistical inferences in the data analyses.*  *Specifically, it points to the application of a propensity score matching- difference in difference (PSM-DiD) method. It also states that “our study proceeds to estimate, for the first time, the intrinsic value (economic cost) of QALYs lost due to a serious injury or illness”* |
| Setting | 5 | Describe the setting, locations, and relevant dates, including periods of recruitment, exposure, follow-up, and data collection | Pages 3, paragraph 3 - Section 2  *The paragraph indicates Australia as the setting and explains the cohort identification year of the study as 2014, with annual follow-ups for five years.* |
| Participants | 6 | (*a*) *Cohort study*—Give the eligibility criteria, and the sources and methods of selection of participants. Describe methods of follow-up  *Case-control study*—Give the eligibility criteria, and the sources and methods of case ascertainment and control selection. Give the rationale for the choice of cases and controls  *Cross-sectional study*—Give the eligibility criteria, and the sources and methods of selection of participants | Page 4, paragraph 3  Page 5, paragraph 1  *The age 15 and injury or illness status eligibility criteria are fully explained.* |
|  |  | (*b*) *Cohort study*—For matched studies, give matching criteria and number of exposed and unexposed  *Case-control study*—For matched studies, give matching criteria and the number of controls per case | Page 5, paragraph 2  The PSM (i.e., one-to-one nearest neighbour matching without replacement) approach used for matching and identifying the unexposed group is detailed in this paragraph |
| Variables | 7 | Clearly define all outcomes, exposures, predictors, potential confounders, and effect modifiers. Give diagnostic criteria, if applicable | Page 3, Section 2.2 and 2.3  *The health-related quality of life measures (outcomes) and serious injury or illness (exposure) variables are defined and explained in these sections.*  *Page 6 paragraphs 1 and 3 discuss control variables with references.* |
| Data sources/ measurement | 8* | For each variable of interest, give sources of data and details of methods of assessment (measurement). Describe comparability of assessment methods if there is more than one group | Page 3, Section 2.1  Page 4, Section 2.3  *Write-ups in these sections show that variables are obtained from the HILDA survey questionnaire. The measurements of these variables are also explained.* |
| Bias | 9 | Describe any efforts to address potential sources of bias | Page 5, paragraph 2 - Section 2.4  Page 6, paragraphs 1& 2  *The use of propensity score matching-difference in differences (PSM-DiD) method and how it addresses potential sources of bias is explained.* |
| Study size | 10 | Explain how the study size was arrived at | Page 5, Figure 1  *Our study uses secondary data, and Figure 1 shows the extracted sample in each wave of the data* |
| Quantitative variables | 11 | Explain how quantitative variables were handled in the analyses. If applicable, describe which groupings were chosen and why | **Variable handling**  ***Page 4, Section 2.2***   - *Explains how the six health-related quality of life measures were obtained from the* SF-36 instrument   ***Page 4, Section 2.3***   - *Explains how serious injury or illness was derived* |
| Statistical methods | 12 | (*a*) Describe all statistical methods, including those used to control for confounding | Page 5, paragraph 2 - Section 2.4  Page 6, paragraphs 1& 2  *The application of the propensity score matching-difference in differences (PSM-DiD) method and how it is employed* to produce the results is detailed in these paragraphs. |
|  |  | (*b*) Describe any methods used to examine subgroups and interactions | This study did not engage in sub-group analysis. |
|  |  | (*c*) Explain how missing data were addressed | Page 6 paragraph 3  *Individuals with missing information on HRQoL during any of the study years were excluded from the analysis, hence we utilised an intention-to-treat analytical approach.* |
|  |  | (*d*) *Cohort study*—If applicable, explain how loss to follow-up was addressed  *Case-control study*—If applicable, explain how matching of cases and controls was addressed  *Cross-sectional study*—If applicable, describe analytical methods taking account of sampling strategy | Page 6 paragraph 3  Page 5 section 2.4, paragraph 1 explains how matching was performed and the metric used to ensure to check the robustness of matching (standardised mean deviation). |
|  |  | (*e*) Describe any sensitivity analyses | Pages 10 to 13—Section 3.4  **Tables 3, 4, 5 & 6**   - *This section is dedicated to sensitivity tests. We separately estimated outcomes for groups with and without secondary exposure to serious injury or illness in the follow-up period.* - *We also tested for possible omitted variable bias and analysed data to account for potential recall bias* |

| Results | | | |
| --- | --- | --- | --- |
| Participants | 13* | (a) Report numbers of individuals at each stage of study—eg numbers potentially eligible, examined for eligibility, confirmed eligible, included in the study, completing follow-up, and analysed | Page 5, Figure 1 and Appendix A   - *Figure 1 shows the extracted sample in each follow-up period.*   Pages 7, paragraph 1—Section 3.1  Pages 7, *Table 1*   - *The table and paragraphs detail the exposed and control samples before and after matching.* |
|  |  | (b) Give reasons for non-participation at each stage | Pages 3 (& 4), paragraph 5 (& 2) - Section 2 |
|  |  | (c) Consider use of a flow diagram | Page 5, Figure 1   - *Figure 1 shows the extracted sample in each follow-up period.* |
| Descriptive data | 14* | (a) Give characteristics of study participants (eg demographic, clinical, social) and information on exposures and potential confounders | Pages 7, paragraphs 1 & 2  Pages 7, *Table 1*  *Summary statistics and associated write-ups on demographic and other variables used in the analyses are provided here.* |
|  |  | (b) Indicate number of participants with missing data for each variable of interest | *Page 7 paragraph 1 (Figure 1 and Appendix A)* |
|  |  | (c) *Cohort study*—Summarise follow-up time (eg, average and total amount) | Page 3, paragraph 3 - Section 2   - *The paragraph explains the cohort identification year of the study as 2014, with annual follow-ups for five years.*   Page 5, Figure 1   - *Our study uses secondary data, and Figure 1 shows the extracted sample in each wave of the data, including follow-ups* |
| Outcome data | 15* | *Cohort study*—Report numbers of outcome events or summary measures over time | Pages 7, paragraphs 1 & 2  Pages 7, *Table 1 and Page 8 paragraph 1 and Appendix B*  *Summary statistics for outcome variables and their associated write-ups are provided here.* |
|  |  | *Case-control study—*Report numbers in each exposure category, or summary measures of exposure |  |
|  |  | *Cross-sectional study—*Report numbers of outcome events or summary measures |  |
| Main results | 16 | (*a*) Give unadjusted estimates and, if applicable, confounder-adjusted estimates and their precision (eg, 95% confidence interval). Make clear which confounders were adjusted for and why they were included | Pages 8, Section 3.2  Tables 2   - The main results from the *propensity score matching-difference in differences (PSM-DiD), which captures the effect of Serious injury or illness on health-related quality of life are reported here. 95% confidence intervals and p-values are also reported.* |
|  |  | (*b*) Report category boundaries when continuous variables were categorized | Appendices C-E  *The age and education categories and details are in this table.* |
|  |  | (*c*) If relevant, consider translating estimates of relative risk into absolute risk for a meaningful time period | N/A  Our study does not report relative risk |
| Other analyses | 17 | Report other analyses done—eg analyses of subgroups and interactions, and sensitivity analyses | Page 9, Section 3.3  **Figure 2**   - *Economic cost of QALY loss due to a serious injury or illness*   Pages 10 to 13—Section 3.4  **Tables 3, 4, 5 & 6**   - *This section is dedicated to sensitivity tests. We separately estimated outcomes for groups with and without secondary exposure to serious injury or illness in the follow-up period.* - *We also tested for possible omitted variable bias and analysed data to account for potential recall bias* |
| Discussion | | | |
| Key results | 18 | Summarise key results with reference to study objectives | Pages 13-15, Section 4  This section provides a discussion of our findings. |
| Limitations | 19 | Discuss limitations of the study, taking into account sources of potential bias or imprecision. Discuss both direction and magnitude of any potential bias | Page 15, Paragraph 2  *This section discusses the limitations of our study.* |
| Interpretation | 20 | Give a cautious overall interpretation of results considering objectives, limitations, multiplicity of analyses, results from similar studies, and other relevant evidence | Pages 13-15, Section 4  This section provides an interpretation by linking findings to the existing literature. It also discusses why our findings differ from those of previous studies. |
| Generalisability | 21 | Discuss the generalisability (external validity) of the study results | Pages 15, Paragraph 3  This is detailed in the concluding paragraph. |
| Other information | | | |
| Funding | 22 | Give the source of funding and the role of the funders for the present study and, if applicable, for the original study on which the present article is based | Page 18  This research did not receive any specific grant from funding agencies in the public, commercial or not-for-profit sectors.  . |

*Give information separately for cases and controls in case-control studies and, if applicable, for exposed and unexposed groups in cohort and cross-sectional studies.

**Note:** An Explanation and Elaboration article discusses each checklist item and gives methodological background and published examples of transparent reporting. The STROBE checklist is best used in conjunction with this article (freely available on the Web sites of PLoS Medicine at http://www.plosmedicine.org/, Annals of Internal Medicine at http://www.annals.org/, and Epidemiology at http://www.epidem.com/). Information on the STROBE Initiative is available at www.strobe-statement.org.
